# Achieving bottom-up ethical, legal, and societal implications and responsible research and innovation in the synthetic biology research community from the Japanese context

**DOI:** 10.3389/fgene.2026.1716198

**Published:** 2026-03-18

**Authors:** Makiko Matsuo, Katsumi Hagino, Saya Kawata, Tomohisa Hasunuma, Kohsuke Honda

**Affiliations:** 1 Graduate School of Public Policy, The University of Tokyo, Tokyo, Japan; 2 Department of Bacteriology, University of Wisconsin-Madison, Madison, WI, United States; 3 Graduate School of Arts and Sciences, The University of Tokyo, Tokyo, Japan; 4 Engineering Biology Research Center, Kobe University, Kobe, Japan; 5 International Center for Biotechnology, Osaka University, Osaka, Japan

**Keywords:** ELSI, RRI, governance, synthetic biology, engineering biology, Japan

## Abstract

Biomanufacturing and synthetic biology are increasingly seen as essential to realizing a global bioeconomy. Within the broader trends in science and technology policy, the emphasis on foresight and addressing societal challenges has been growing. Addressing ethical, legal, and social implications/issues (ELSI) has become a prerequisite for responding to these trends in several countries, including Japan. This paper focuses on a specific aspect of ELSI, rulemaking, which is attracting increasing attention in the policy context. It highlights the lack of sufficient bottom-up initiatives from the academic research and development (R&D) community in this area and identifies three key areas for action that should be considered: (1) Advancing R&D informed by technological trends as well as policy and societal developments, (2) engaging in proactive deliberation to ensure safety and security, and (3) contributing to discussions on standardization. Moving forward, these recommendations must be elaborated on through discussions with universities, academic societies, the government, funding agencies, industries, and other stakeholders.

## 1 Introduction

### 1.1 Background: trends in science and technology policy discussions—Foresight and the orientation toward solving social issues

Since the Organisation for Economic Co-operation and Development (OECD) proposed the concept of the bioeconomy (OECD, 2009), over 50 countries have now actively developed strategies for creating them (Gardossi et al., 2023). In the face of growing concern regarding global warming, the demand to transition from current fossil fuel–based production and industrial systems—which emit large amounts of CO_2_—to more sustainable, bio-based alternatives has increased. Within this context, expectations for bio-based manufacturing and its foundational technology, synthetic biology, are rising (McKinsey Global Institute, 2020; Hodgson et al., 2022). Japan is promoting a bioeconomy strategy aimed at creating a market worth approximately 100 trillion yen domestically and internationally by 2030. Biomanufacturing based on synthetic biology is positioned as a central pillar of this strategy. In the fiscal year 2022, a large-scale budget of around 1 trillion yen was allocated to national projects such as the Green Innovation Fund Project, the Biomanufacturing Revolution Promotion Project, the Green Technologies of Excellence (GteX) Program, the Project for Developing Biopharmaceutical Manufacturing Sites to Strengthen Vaccine Production, and the Strengthening Program for Pharmaceutical Startup Ecosystem (Cabinet Office, 2024). As these policies unfold, the projected market size is estimated to reach USD 19,145 million in Japan (USD 329,655 million globally) by 2030 (Ministry of Economy Trade and Industry, 2024). Furthermore, under the administration of Prime Minister Takaichi, who came into office in October 2025, synthetic biology and biotechnology have been designated as a priority.

Two trends are evident in recent science and technology policies. The first is the demand for embedding forward-looking policymaking. The emergence of various critical emerging technologies—such as biotechnology, artificial intelligence (AI), and quantum technology—has created a “pacing problem,” where the speed of technological development outpaces oversight (Marchant et al., 2011; Marchant et al., 2013). To avoid this, the demand for anticipatory governance^1^, which means proactively understanding the possible societal impacts of technology in advance and preparing for them, has been increasing (Marchant et al., 2011; OECD, 2020). Another emerging trend in science and technology policy is the growing emphasis on addressing so-called “grand challenges.” Policymakers now expect technological development to contribute to solving complex societal issues, such as global warming, leading to increased interest in mission-oriented innovation policy (MOIP) (Wanzenböck et al., 2020; OECD, 2021; Matsuo, 2024). To address societal challenges effectively, a fundamental transformation of societal values and industrial structures is imperative. This necessitates a comprehensive examination of technology’s role in a broader context. This means that, in addition to understanding the technology itself, we must also examine its societal impact, including the interaction between technology and society, and the underlying social context. Synthetic biology, the focus of this paper, is touted as contributing to the bioeconomy, circular economy, and nature positivity (Shapira et al., 2022, p78). However, from both responsibility and accountability perspectives, it is essential to explain how it specifically contributes to all these social needs.

Therefore, to advance both anticipatory governance and the MOIP, it is crucial to proactively examine the societal impacts of technology and shape the direction of technology. The concept of technology assessment (TA) proves useful in this context (OECD, 2023b). Although there is no commonly agreed definition, TA is described as *“a wide category encompassing an array of policy-analytic, economic, ethical, and other social science research that attempts to anticipate how research and research-based technologies will interact with social systems”* (Guston and Sarewitz, 2002). In practice, TA takes diverse forms and incorporates a variety of elements, including environment, health, and safety (EHS) studies Ethical, Legal, and Social Implications/Issues (ELSI) studies, risk perception and communication studies, and Responsible Research and Innovation (RRI) (Grunwald, 2018). As is well known, ELSI originated in the United States within the framework of the Human Genome Project, where a portion of the budget was allocated to examining ELSI. RRI is a more recent concept developed as the necessary components and frameworks (Stilgoe et al., 2013) evolved alongside policy initiatives promoted by the European Commission. Both approaches share a common focus on responding to the societal impacts that may arise in the future at the interface between technology and society. As such, they are often used interchangeably—especially in Japan, where ELSI and RRI are frequently discussed together (see for instance, JST CRDS, 2023). However, it is important to note that their historical origins, theoretical backgrounds, and practical applications differ. While this paper does not delve into those differences, there is a research gap in understanding them, and studies (Robinson et al., 2023) aimed at clarifying these distinctions are important.

### 1.2 ELSI consideration and responsibility—from top-down to bottom-up collaboration

Governance refers to all the processes of governing (Bevir, 2012). The shift from “government to governance” (Rhodes, 1996)—from a traditional, centralized government with strong enforcement mechanisms to a more decentralized, networked form of governance involving diverse actors, including the private sector—is particularly significant in the governance of emerging technologies.

Traditional technology governance has primarily taken the form of a top-down approach led by central government authorities, wherein measures to address societal impacts were naturally considered within the policy process; in particular, the legal aspects of ELSI were regulated at the national level, primarily by the relevant ministries and agencies. However, the limitations of such policy developments are well-recognized. First, while such top-down policies have strengths in terms of enforcement, bureaucratic procedures tend to be slow and make responses time-consuming. Given the rapid pace of technological advancement, policymakers face inherent limitations in adequately grasping all the impacts of cutting-edge scientific and technological trends or in promptly evaluating their associated ELSI. Second, existing ELSI review processes centered on government expert advisory councils often lack comprehensive perspectives, particularly in terms of incorporating and prioritizing the viewpoints of society and citizens who might be directly affected by the technology, as observed in the discussion of increasing recognition of the emphasis on the democratization of science and technology and deliberative processes. Consequently, bottom-up deliberation involving diverse stakeholders is being considered increasingly necessary. Bottom-up approaches allow rapid deliberation once consensus is reached and allow for immediate initiation of voluntary action. However, this approach has inherent limitations, particularly regarding enforceability and legitimacy.

Therefore, in governing emerging technologies, collaboration between various actors that balances the above-mentioned advantages and disadvantages of the top-down and bottom-up approaches is essential. Despite the growing need for bottom-up ELSI deliberations, a significant challenge remains: R&D entities themselves often lack awareness of the importance of engaging with safety and security, and ELSI considerations and related initiatives are insufficiently developed. Research communities, particularly those within academia, have traditionally been passive in rule-making processes. However, achieving anticipatory governance requires the proactive involvement of all actors engaged in technological development (OECD, 2020), including the academic and research communities.

### 1.3 Purpose and structure of this paper

Therefore, this paper focuses on the research and development community, namely, academia, one of the essential bottom-up actors whose role in considering ELSI has been insufficiently explored in conventional research, and aims to discuss the ELSI these communities should address, as well as the policies and ecosystem consideration for policymakers that could enable such consideration, with particular attention to the Japanese context. In Japan, there is generally strong trust in government-led, top-down measures, and once decisions are made, they tend to be highly effective. At the same time, there is considerable reliance on administrative bodies for rulemaking and safety measures. Japan’s administrative system emphasizes the development of generalists and involves frequent short-term rotations across various departments. While this approach facilitates horizontal coordination, it can be a weakness when addressing governance challenges posed by emerging technologies—such as the need for continuous updates, technical expertise, dealing with uncertainty, and long-term strategic thinking. Therefore, strengthening bottom-up approaches, which are currently lacking, could complement top-down mechanisms and enhance overall governance capacity.

In Section 2, we outline the development of ELSI considerations in Japan’s science and technology policy, how ELSI is addressed in its biotechnology policy and bio-related research areas, and the challenges it faces. Section 3 explores specific ELSI items that the R&D community should actively consider in future biomanufacturing policies in Japan. Finally, Section 4 concludes the paper with actionable recommendations.

## 2 Development of ELSI and its challenges in the Japanese context

### 2.1 Development of ELSI in Japan’s science, technology, and bioeconomy policies and biotechnology-related research areas

In Japan, there is a growing tendency to emphasize the integration of ELSI^2,^ ^3^ considerations into science and technology policies (Shiroyama and Matsuo, 2024). ELSI has been explicitly mentioned from the third Science and Technology Basic Plan (2006–2011) and is emphasized in the current sixth Science and Technology Basic Plan (2021–2026). The Center for Research and Development Strategy (CRDS) of the Japan Science and Technology Agency (JST), a funding agency under the Ministry of Education, Culture, Sports, Science, and Technology (MEXT), has published numerous strategic proposals (JST CRDS, 2023) and reports (JST CRDS, 2019; JST CRDS, 2021; JST CRDS, 2022), seeing ELSI as a strategically critical component of national research and development and recognizing it as a key issue to be addressed through science and technology policy.

There has also been a movement to incorporate ELSI into national R&D programs and projects. For example, the Moonshot R&D Program, a national program led by the Cabinet Office, has established an ELSI Subcommittee^4^ to provide cross-disciplinary ELSI support for each project. Some projects in this program require ELSI to be positioned within the research project design at the application stage^5,^ ^6,^ ^7,^. Furthermore, several projects have developed guidelines for ELSI^8^. For example, the project under Goal 1, Liberation from Physical Constraints through Enhancement of Physical and Perceptual Capabilities, has established a committee to create Brain-Machine Interface (BMI) usage guidelines^9^. It is developing an ELSI-related guidebook (Advanced Telecommunications Research Institute International (ATR), 2024; Advanced Telecommunications Research Institute International (ATR), 2025) while pursuing international collaboration with organizations such as the OECD.

In biotechnology-related national policies, ELSI is positioned as an important element within the Integrated Innovation Strategy and the Bio Strategy in Japan^10^. A recent example^11^ at the specific research project level is the JST Strategic Basic Research Program (CREST/Sakigake) research area Genome Programming - Large-Scale Genome Synthesis and Cell Programming. In this project, applicants were encouraged from the initial stage of public funding calls to consider ethical, legal, and social issues anticipated in the context of future practical application of technology. JST’s Research Institute of Science and Technology for Society (RISTEX) established the Genome Ethics Study Group which comprises diverse stakeholders including researchers in humanities, social sciences, and natural sciences, industry representatives, and practitioners. This group also collaborates with the project to conduct other activities^12^. For example, the group held online seminars led by its members, created ELSI issue maps^13^, and conducted several case studies^14^—group interviews and workshops in CREST researchers’ labs—to identify key ELSI-related issues. Another notable case from the JST research project where academia played a leading role is JST’s Co-creation of Molecular Robot ELSI and Real-time Technology Assessment Research (2017–2020). This project formulated the Ethical Principles for Molecular Robotics (Molbot) (Yoshizawa et al., 2018; Komiya et al., 2022). Subsequently, at the second Annual Molecular Robotics Conference in 2019, the Ethical Principles for Molecular Robotics Technology Version 1.2 (Revised 14 March 2019) was released as the consensus of the domestic molecular robotics research community (Konagaya Laboratory, 2019; Komiya et al., 2022). Other academic initiatives include the Japanese Society for Cell Synthetic Research, which continuously organizes sessions at annual conferences to facilitate discussions on science, technology, and ELSI.

### 2.2 Challenges in the practical implementation of ELSI and the growing focus on the legal aspect of ELSI

While the policy emphasis on ELSI is increasing and several notable initiatives have been made, these efforts remain largely sporadic. ELSI activities have not yet been properly embedded within policy^15^ or R&D processes, and they remain insufficiently internalized within the R&D community.

A particularly challenging point in the practical application of ELSI is the lack of shared understanding, both at the policy level and within the research community, of the specific activities that constitute ELSI-related work. For example, although this may not be unique to Japan, ELSI tends to be narrowly interpreted. In some cases, this is understood as issues of research integrity, misconduct, or fairness (Tanaka et al., 2022). In other cases, it means bioethics in the biomedical field^16^, whereas in the agricultural domain, it is associated with social acceptance issues^17^. Interpretations reflect each application area’s characteristics and contribute to the narrow understanding of ELSI (narrowly defined ELSI). In practical cases of ELSI and RRI, there is no uniform “correct” approach; it must inevitably be case-by-case. While development tools listing considerations to facilitate ELSI deliberation can be useful to a certain degree^18,^ ^19,^ ^20^, such tools cannot be generalized or reduced to simple checklists. Factors to consider for ELSI vary depending on the characteristics of the technology, its level of maturity, technology readiness level (TRL), and the field of application. Consequently, RRI considerations are inherently diverse (often referred to as the plurality of RRI), and a tailored approach is often advocated (Smith et al., 2019).

Furthermore, as discussed later in this paper, the increasing needs to address “grand challenges”—the mission-oriented nature of science and technology policy—along with changes in the social landscape, such as heightened geopolitical tensions, are expanding the issues to be analyzed in terms of ELSI and accountability perspectives. Consequently, there is an increasing trend in the scope of matters considered by ELSI (Hishiyama, 2022). Particularly emphasized in recent years from the ELSI and RRI perspective is the aspect of rulemaking^21^ ^,^ ^22^.

Traditionally, rulemaking was considered the domain of policymakers, but there is now discussion about embedding it at an earlier stage, specifically within R&D. The JST CRDS outlines the following three proposals in its Strategic Proposal (JST CRDS, 2023): (1) strengthening hub functions connecting rule/norm formation activities with R&D work; (2) embedding ELSI/RRI practices in R&D programs promoted under the national Science, Technology, and Innovation (STI) policy; and (3) securing diverse talent/human resources to bridge R&D and rule/norm-making.

## 3 Future initiatives for the synthetic biology research community in developing ELSI with rule formation in mind

In light of the policy needs and background addressed in the previous section, the following three areas are identified as key activities that the R&D community should now focus on: (1) Advancing R&D informed by technological trends as well as policy and societal developments, (2) engaging in proactive deliberation to ensure safety and security, and (3) contributing to discussions on standardization.

### 3.1 Advancing R&D informed by technological, policy, and societal trends

The first is the need to engage in R&D based on a thorough understanding of future technological trends and policies as well as social developments. Basic science does not automatically lead to applied science nor does it lead to the social introduction of that technology; science and technological innovation are mutually interlinked activities (Brooks, 1994). Therefore, the societal impacts that may arise in the future must be proactively considered. Activities that broadly capture such future signs and nascent trends are termed horizon scanning. The importance of horizon-scanning in implementing anticipatory governance in policy formation and technology development governance is highlighted. Similarly, its significance is strongly emphasized for responding to future bioproducts (NASEM, 2017) and advancing the bioeconomy (NAS, 2020). Examples of horizon-scanning in academia include Kemp et al. (2020) and Wintle et al. (2017). In Japan, entities such as the National Institute of Science and Technology Policy (NISTEP), MEXT, JST CRDS, and New Energy and Industrial Technology Development Organization (NEDO), Ministry of Economy, Trade and Industry (METI) also conduct horizon scanning activities. Project-based initiatives have also undertaken such activities. For instance, within the JST Green Technology of Excellence (GteX) program, researchers engaged in mini-horizon scanning roundtable discussions, enabling them to identify and discuss possible future technological and societal trends (Matsuo et al., 2024).

Bringing such forward-looking horizon scanning and backcasting activities into the R&D process enables R&D communities to be aware of the possible challenges ahead in the societal implementation of technology. For instance, realizing a biofoundry, which is now a major policy focus in several countries, requires effective integration of the elemental technological components involved in the Design-Build-Test-Learn (DBTL) cycle. Furthermore, scaling up engineered host organisms for large-scale production is becoming increasingly challenging. Particularly in this field, with several new entrants and actors in different disciplines and sectors (biology, software, robotics, etc.), ecosystems where a single entity handles the entire technology development process from upstream to downstream are becoming less common (Clarke and Kitney, 2020). Therefore, it is crucial to design at an early R&D stage by adopting a backcasting approach that considers the constraints of the downstream stages of the technology development process.

In the aforementioned JST GteX program discussion, along with debates on technological trends, such as digitalization, lab automation, and the introduction of AI, social impacts and challenges were also addressed by the researchers. For instance, because the project’s mission is to reduce CO2 emissions, the discussion emphasizes the necessity of how the development of technology relates to environmental and energy issues. This led to their understanding that research must be designed considering the availability of domestic raw materials and bio-resources, along with various other points raised, such as standardization and human resource development. Indeed, ensuring feedstock and raw materials is a policy challenge not only in Japan but also in many other countries, making proper alignment between the R&D community and policy crucial. Beyond these discussions, broader landscape-level shifts—such as the growing “securitization” of various issues driven by heightened economic security concerns and geopolitical tensions and the resulting challenges to multilateralism (OECD, 2023a)—may also have a potential influence on research activities. Given these landscape-level influences, it is vital for researchers to remain aware of how such changing landscape-level trends can influence the sustainability and competitiveness of their R&D and consider proactively responding to these changes.

Therefore, the R&D community must envision and anticipate how fundamental research can be scaled up and introduced into society. To achieve this, they need to position their work within a broader understanding of societal demands, needs, values, rules, and the evolving social environment, including socio-geopolitical trends. Such considerations should be pursued not only at the project level but also within academic societies, both within the country and internationally.

### 3.2 Engaging in proactive deliberation to ensure safety and security

The second aspect is a bottom-up consideration to ensure safety and security. Ensuring safety and security are fundamental prerequisites for the social introduction of technology. Safety refers to the measures implemented to prevent the accidental release or unintended exposure to biological agents and toxins. Security refers to measures implemented to prevent the loss, theft, misuse, diversion, intentional release, unauthorized access, possession, or transfer of biological agents, toxins, and related resources (WHO, 2024). The key difference between the two is whether the event is accidental or intentional. Numerous international organizations and frameworks already exist, and rule formation is progressing. In the biosafety domain of Living Modified Organisms (LMOs), the Convention on Biological Diversity (CBD) addresses environmental and ecosystem protection when LMOs are released into the environment. For human safety, Codex Alimentarius Commission guidelines exist to enable Genetically Modified Organisms (GMOs) to be consumed as food. The concepts developed within these forums (such as *familiarity* with environmental impact assessments, *comparative approaches*, and *case-by-case* responses) have been incorporated into the laws, regulations, and management systems of major developed countries. In Japan, impacts on biodiversity based on agreements from the above international organizations are managed by the Ministry of the Environment and related ministries under the Cartagena Law, whereas safety assessments for consumption as food are managed under the Food Sanitation Act and the Basic Act on Food Safety (Matsuo and Tachikawa, 2022). Whereas the Geneva Protocol and Biological Weapons Convention (BWC) address security from a traditional security perspective, forums and international organizations such as the World Health Organization (WHO), Food and Agriculture Organization of the United Nations (FAO), and World Organization for Animal Health (WOAH) address it from a public health perspective, from human, agricultural, and livestock aspects, respectively.

In Japan, pathogens are properly managed at Biosafety Levels (BSLs) according to the Infectious Diseases Control Law^23^. Furthermore, several technologies possess dual-use potential, meaning they can be used for both peaceful and malicious purposes (Miller and Selgelid, 2007). In Japan, the Science Council of Japan revised its Code of Conduct for Scientists—Revised Edition in 2013 to include provisions on dual-use issues (Science Council of Japan, 2013). Following this, it issued recommendations on dual-use concerns in pathogen research (Science Council of Japan, 2014). Given the multifaceted nature of security—encompassing traditional national security, public health security, research integrity, and more—it has historically been addressed within separate domains rather than being fully integrated into science and technology policy. However, influenced by recent geopolitical developments, the Basic Plan for Science, Technology, and Innovation, scheduled for 2026, is expected to emphasize the linkage between science, technology, and national security—a significant policy shift. How to embed these considerations into science and technology policy and research promotion remains a challenge.

Compliance and adherence to the laws, regulations, and international norms established through these forums and adopted by individual countries are fundamental prerequisites for advancing the research and responsibilities of those engaged in R&D. However, the compliance to existing laws and regulation is not enough for ensuring safety and security of emerging technology. Below, we would like to shed light on the legal aspect of the ELSI issue that may not be sufficiently covered by existing forums or regulatory frameworks due to rapid technological advancement, which can give rise to “pacing problems”, where governance and oversight find it difficult to keep pace with the speed of technological development. Such issues include the potential risks posed by xenobiology and convergence with AI, as discussed below.

To provide an example, in the field of safety, safety assessments have traditionally been based on comparisons with *conventional counterparts* (this approach underpins safety assessments in every country). While this comparative approach still remains a useful method for most of the technologies that are currently being developed, it has been suggested that it may not function for technologies emerging in the future (NASEM, 2017; EFSA Panel on Genetically Modified Organisms et al., 2022). For instance, in xenobiology, cells can be developed using amino acids or alternative DNA bases that are not found in nature, potentially creating entities for which no existing comparators exist. Furthermore, advances in genome editing technology have enabled the insertion of genes from multiple species, raising questions regarding the applicability of the traditional comparative approach to safety assessment. Thus, when introducing new technologies, whether existing frameworks are sufficient or whether new safety assessment methodologies should be developed to address the potential associated risks must be considered. Developers must also integrate safety considerations throughout the R&D design process and collect and retain related data for consideration (EFSA Panel on Genetically Modified Organisms et al., 2022). Another area in which traditional regulatory science for safety assessments is anticipated to be insufficient is the development of Engineered Microbes for Environmental Release (EMERs). For a long time, genetically engineered microorganisms (GMMs) were intended for contained use; however, recent developments are intended for environmental release, and examples of potential applications include bioremediation and biosensor technologies (Marken et al., 2024). The development of intentional release of GMMs in the open environment must be accompanied by the establishment and clarification of safety assessment methods.

In the field of security, rapid technological advances have prompted changes to reconsider scientific approaches for ensuring security. For instance, biosecurity levels have traditionally been categorized based on lists reflecting the inherent risks of the pathogens themselves. However, technological advances now enable the referral of security-related functions to agents not previously considered (e.g., novel organisms created via xenobiology) and can potentially evade traditional detection methods based on PCR or DNA sequencing (NASEM, 2018), suggesting that traditional list-based management may become ineffective.

Advances in AI can strengthen the means of addressing these safety and security challenges. For instance, regarding the aforementioned lack of comparators for safety assessments, AI might enable the consideration of extrapolation through complex, similar comparisons. Furthermore, tools such as AlphaFold 3 (Abramson et al., 2024) and RoseTTAFold All-Atom (Krishna et al., 2024) enable protein folding and functional prediction, making it possible to pursue safer structures. Conversely, however, they can amplify the risks. Currently, it is technically difficult to develop completely new (*de novo*) malicious pathogens using AI (EBRC, 2025; NASEM, 2025). However, screening can be avoided by modifying known pathogens to increase pathogenicity (NASEM, 2025) or by creating variants with low sequence homology that have functions equivalent to those of highly virulent wild-type strains (EBRC, 2025). Globally, discussions on how to screen sequences of concern (SoC) to address biosecurity are taking place within forums and organizations, such as the consortium of genome synthesis companies and the International Gene Synthesis Consortium (IGSC), as well as within the International Biosecurity and Biosafety Initiative for Science (IBBIS) and Engineering Biology Research Consortium (EBRC).

Both cases highlight the fact that, as technology advances and methodologies diversify, the traditional management frameworks, concepts, and approaches that were previously assumed to provide sufficient safety and security may no longer allow for uniform or one-size-fits-all risk assessment. This necessitates a risk-based case-by-case examination. Risk-based and case-by-case responses require more sophisticated and careful consideration from decision makers. This necessitates foundational information and insights from the field held by researchers and developers. Policymakers cannot understand the real impact of these cutting-edge upstream technologies, and scientists cannot predict how quickly something can be developed.

Therefore, the R&D community must work together to undertake the following activities as part of their research and development: (1) Implement development designs that incorporate safety and security considerations from the design phase (incorporating the principle of Safety/Biosecurity *by Design*) and consciously collect and accumulate data from the development stage. Furthermore, the necessity of these activities should be incorporated into education and curricula and discussed at academic conferences; (2) Develop technologies related to biological containment that technically reduce risk, such as “kill switch” technology (Stirling et al., 2017) that achieves target organism death through environmental changes or “recoding technology” (Lajoie et al., 2013) that confines modified organisms by requiring artificial amino acids not found in nature for survival through genetic reconstruction. Such technological containment measures should be recognized as an essential set of technologies for mitigating risks in open systems. (3) When facing technologies with potential concerns or their impacts, researchers themselves should issue warnings (e.g., Adamala et al., 2024a), and the entire R&D community should engage in bottom-up discussions with relevant stakeholders to determine appropriate responses to the question of what conditions are needed to allow research to proceed. Where necessary, efforts should be made to engage governments and international organizations. Similarly, dual-use issues where clear boundaries are difficult to define, including challenges such as weighing the benefits and risks of gain-of-function (GoF) research, the appropriate level of information disclosure, and the extent to which research freedom should be guaranteed, are subject to ongoing discussion.

### 3.3 Contributing to discussions on standardization

The third area for bottom-up initiatives in need of an R&D community is standardization. Standardization can enhance interoperability, reproducibility, and efficiency across the research, development, and commercialization stages. It is also expected to streamline regulatory approval processes, support market entry for small and medium-sized enterprises, and improve biosafety (Pei et al., 2022; Freemont et al., 2024; Robinson and Nadal, 2025). The need for standardization is highlighted in numerous policy documents, including Japan’s Bioeconomy Strategy, a report submitted by the National Security Committee on Emerging Biotechnology (NSCEB) at the US Congress (NSCEB, 2025), and the UK’s National Vision for Engineering Biology (Department for ScienceInnovation and Technology, 2023). Discussions are also underway within organizations, such as the Global Biofoundry Alliance (GBA), which involves over 40 biofoundries, primarily from academia, and the U.S. public-private platform EBRC (Freemont et al., 2024).

However, the term standardization is often used to mean various things, and its scope encompasses a wide range of things. Therefore, this section organizes the standardization necessary for engineering biology practice into three main categories: (1) standardization of foundational technologies (Data, Terminology, Methodology); (2) standardization of biomanufacturing systems (Chassis, Scale-up/Scale-out, Biomass feedstocks), and (3) standardization for evaluation and societal implementation (sustainability assessment, Life Cycle Assessment (LCA), etc.).

In the first category, the standardization of foundational technologies and data standardization (Borgman, 2012; Gonzalez and Peres-Neto, 2015) is crucial. It is important to follow guidelines such as the FAIR principles (Wilkinson et al., 2016; Gonzalez Soltero et al., 2024) for data publication, the Minimum Information Checklist (Lee et al., 2008; Bustin et al., 2009), and the ISA Metadata Framework (Johnson et al., 2021), a framework for describing metadata such as experimental conditions and objectives. Regarding terminology and vocabulary, Synthetic Biology Open Language (SBOL) is now established as a machine-readable format for gene design information (Galdzicki et al., 2014; McLaughlin et al., 2020), while Gene Ontology (GO) has been developed as a common vocabulary for describing gene functions (Hill et al., 2013; The Gene Ontology Consortium, 2021). Such common languages improve communication efficiency among researchers and enhance literature search accuracy (Peccoud et al., 2011; Awaysheh et al., 2018). However, several challenges remain to be resolved. These terms are inherently dynamic and require re-evaluation and updates when new technologies or discoveries emerge (e.g., genome editing). Additionally, international harmonization of terminology is difficult to achieve, as non-English-speaking regions require translation into local languages for its use (Freemont et al., 2024). Standardizing methodologies related to experimental protocols, manufacturing processes, and quality control procedures can reduce inter-experiments variability and improve process transfer success rates (Freemont et al., 2024; Parks et al., 2017; Schilling et al., 2008; Lorenz et al., 2019). Japan also has initiatives related to standardization at the level of reference resources and measurement. For example, the NITE (National Institute of Technology and Evaluation) Biological Resource Center (NBRC) culture collection provides lists of microbial test strains specified in ISO, Japanese Industrial Standards (JIS), and the Japanese Pharmacopoeia for use in standardized examinations^24^. In measurement, the National Metrology Institute of Japan (NMIJ) produces and supplies certified reference materials for chemical and bioanalytical measurements; examples include DNA solutions for quantitative analysis (NMIJ CRM 6205-a, 2023; Shibayama et al., 2019) and clinical chemistry/bioanalysis materials such as C-reactive protein solution (NMIJ, CRM 6201-c, 2024) and C-peptide (NMIJ CRM 6901-c, 2025).

Moreover, databases containing biological information, such as gene sequences, protein data, and metabolic pathways, are indispensable. These databases are maintained by national and regional institutions, such as NCBI^25^ and EMBL^26^, as well as by universities and individual laboratories, with KEGG (Kanehisa et al., 2000) and GTDB (Parks et al., 2018) being notable examples. However, the management of these databases, including their curation and validation, requires substantial time and financial resources. Interoperability between databases remains a challenge because of differences in data formats. Therefore, laboratories should consider adopting compatible formats when creating new databases. It would be helpful to establish clear standards and principles in academic journals and research communities to promote harmonization in database publication and usage. Therefore, the standardization of these aspects requires consideration. The standardization of the DBTL, a new technology development cycle in synthetic biology, is particularly important (Holowko et al., 2021; Van Lent et al., 2023), and entities such as the ISO are also advancing standardization in related fields and technologies. For example, ISO/TC 276 develops standards that support the build/test (analytical methods/processes) and learn (data integration/traceability) phases of the DBTL cycle in a cross-cutting manner (ISO, 2020). ISO/TS 23494 (Providence Information Model) enhances data comparability and reusability, contributing to the efficiency of the design learning loop (ISO, 2023). Additionally, GBA provides access to standardized protocols (Hillson et al., 2019). At the community level, the iGEM operates the Registry of Standard Biological Parts (Shetty et al., 2008) and promotes the adoption of BioBricks (iGEM Foundation, 2025), continuously fostering the sharing of part definitions and standardizing the assembly procedures (iGEM, 2024). If standardized protocols are incompatible with existing laboratory equipment, research activities can be hindered. Therefore, it is essential to advance the discussion on infrastructure development and standardization in parallel. Attempting to advance standardization requires a significant initial investment (Bobier et al., 2024), and some point out that continuous support through industry-academia-government collaboration is essential (FAS, 2006).

The second aspect is the standardization of biomanufacturing systems. One of the topics discussed in this regard is the standardization of the chassis. A chassis is a host organism optimized for a specific purpose. Standardizing its characterization, performance benchmarking, and safety assessment enables improved reproducibility between experiments and shortens development times (Garner, 2021; Ordozgoiti, 2021). Driven by the growing interest in versatility, related projects are currently underway in Japan. For example, the JST GteX program aims to construct “basic cells” equipped with essential standardized functions for biomanufacturing. The standardization has progressed for certain chassis, such as *Escherichia coli* for prokaryotes (Adams, 2016; Hamese et al., 2023), *Saccharomyces cerevisiae* for eukaryotes (Goold et al., 2025), and CHO cells for mammals (Kaas et al., 2015; Reinhart et al., 2019; Cordova et al., 2023). However, establishing predictable and controllable standards is challenging because biological responses fluctuate owing to subtle variations in culture conditions (Szymanski and Henriksen, 2022). To address this challenge, reconstituted artificial cells incorporating biomolecules have recently attracted attention as a next-generation chassis. However, their lack of self-replicating capability is a significant technical barrier to practical implementation (Adamala et al., 2024b; Giaveri et al., 2025). Other topics for the standardization of production systems include standardization related to scale-up and scale-out. This involves maintaining key process parameters during the transition from laboratory to industrial scale and suppressing variations in the target product yield (Delvigne and Noorman, 2017). Examples of company practices include implementing controls via cloud-based systems, such as Culture Biosciences’ cloud bioreactor system (Culture Biosciences, 2020; Culture Biosciences, 2025) and Ginkgo Bioworks’ automated lines (Melody, 2016; Ginkgo Bioworks, 2025). The digital twin technology, which uses simulations to examine scaled-up challenges, is also advancing (Zobel-Roos et al., 2020). However, large-scale bioreactors face unpredictable local malfunctions owing to the nonlinearity of physical phenomena (Delvigne et al., 2017), leading several companies to abandon development in the Valley of Death. Associated economic barriers are also significant (Kampers et al., 2022). Furthermore, laboratory-scale work is typically conducted at a small scale. Differences among equipment (Ding et al., 2024) and the inability to measure and control the parameters required for scale-up in real time (Bellani et al., 2020) create a gap between technological seeds from academia and scale-up, even as standardization progresses on the industrial side.

Additionally, the standardization of biomass feedstock, inputs for production systems, is crucial. This includes unified measurement methods, quality standards, and safety assessments for biomass feedstocks, such as compositional analysis, physicochemical property evaluation, and determination of biological conversion suitability (Freemont et al., 2024). Standardization efforts are advancing through ASTM^27,^ ^28,^ ^29,^ ^30,^ ^31^ and ISO^32,^ ^33,^ ^34^. However, biomass feedstocks are inherently difficult to standardize because of their compositional variability and heterogeneity influenced by origin, harvest time, and weather conditions (Bulsink et al., 2025).

Finally, to standardize the evaluation and societal implementation from the perspective of promoting the bioeconomy, quantifying the environmental impact of synthetic biology products and processes and comparative evaluation with fossil fuel-based alternatives are required (Curran, 2013). Recently, particular attention has been paid to sustainability assessments. One such method, LCA, has an established framework compliant with ISO 14040/44 (ISO, 2006). However, one study found that the majority of 83 bioeconomy-related LCA studies published between 2006 and 2021 were non-compliant with ISO standards (Talwar and Holden, 2022). This highlights the challenges of improving LCA methodologies and creating incentives for compliance. In Japan, efforts are underway to mandate LCA for new R&D projects (NEDO, 2022)^35^ The EU also positions sustainability as a key driver of the bioeconomy^36^. However, the application of LCA to biomanufacturing presents numerous challenges^37^, including the difficulties of direct comparison owing to the differences in functional units between studies (Saavedra Del Oso et al., 2023).

Beyond the challenges discussed above, there are also common structural issues: limitations in prediction accuracy stemming from the complex nature of biological systems (unlike chemicals; Beal et al., 2020), effectiveness constraints due to the temporal gap between technological innovation and standardization (Lorenz et al., 2019), and difficulties in achieving global harmonization due to implementation disparities between regions (Robinson and Nadal, 2025).

Considering the above, it may be worthwhile for the research community to reflect on the following activities regarding standardization as part of its ongoing efforts: (1) making efforts to promote interdisciplinary education and exploring ways of integrating topics related to standardization (e.g., data standards (such as FAIR-compliant data sharing), quality control, and foundational knowledge of LCA) into graduate curricula. Such efforts in early stage training could help foster a deeper understanding among researchers regarding the need for standardization from the outset of their work; (2) Recognizing that a certain level of standardization is necessary for the development of the entire research field, particularly for the reproducibility and sharing of results between laboratories. It is important to further develop and promote bottom-up protocol sharing between laboratories. This can be achieved through platforms such as the STAR protocol^38^ journal for sharing experimental protocols, JOVE (video-based)^39^, and shared repositories, such as protocol.io^40^. Additionally, harmonizing database formats and structures should be considered as a part of these efforts. Furthermore, while information sharing is essential for standardization in R&D, academia is generally less resistant to information disclosure than industry. Therefore, academia can contribute to areas in which industries find it difficult to engage; and (3) In contrast, for academia pursuing originality and novelty, the very act of standardization and unification can be a source of tension. Consequently, the specific areas that can be accepted by the academic research community must be identified. In particular, as mentioned above, if the equipment in a laboratory that is essential for conducting research activities does not conform to unified standards, it can lead to cost issues and problems with research continuity. Therefore, after mapping the overall landscape of areas requiring standardization, it is necessary to explore areas for collaboration through projects and domestic and international academic communities. When a consensus can be reached, consideration should be given to promoting adoption by establishing conditions for academic societies or journal paper acceptance.

## 4 Conclusion—actionable recommendations and discussions

As described in this study, biomanufacturing and synthetic biology are increasingly seen as essential to realizing a global bioeconomy. Within the broader trends in science and technology policy, there is an increasing emphasis on foresight and addressing societal challenges. Addressing ELSI has become a prerequisite for responding to these trends in many countries, including Japan. This paper focuses on the specific aspect of ELSI, rulemaking, which is attracting increasing attention in the policy context. It highlighted the lack of sufficient bottom-up initiatives from the academic R&D community in this area and identified three key areas for action that should be considered: (1) Advancing R&D informed by technological trends, as well as policy and societal developments; (2) engaging in proactive deliberation to ensure safety and security; and (3) contributing to discussions on standardization (see Table 1).

**TABLE 1:** Bottom-up ELSI considerations in the context of rule-making.

Areas in need of bottom-up engagements of the researcher community	Actionable recommendations
(1) Advancing R&D informed by technological, policy, and societal trends	The research community should actively engage in forward-looking activities such as horizon scanning to understand technological trends, societal needs, values, and environmental factors surrounding the technology. Research activities should be conducted with such insights, consciously identifying challenges through backcasting with social implementation in mind. Such consideration should be pursued at the project level, academic society level, and international level
(2) Engaging in proactive deliberation to ensure safety and security	(i) Implement safety/security *by design* from the development stage, ensure data are appropriately acquired and stored from the outset, and develop regulatory science. Incorporate these activities into education and curricula and discuss them within academic societies (ii) Engage in technological development to enhance safety and security, such as developing biological containment technologies that reduce risks through technical means; and (iii) when encountering potential concerns or negative impacts, researchers themselves should raise concerns. The entire research and development community should engage in discussions with relevant stakeholders to address the issues. It is also important to engage with government agencies and international organizations as necessary
(3) Contributing to discussions on standardization	(i) Integrate data standards (e.g., FAIR), quality control, and LCA basics into graduate curricula and provide early training in standard-compliant research practices (ii) Promote bottom-up sharing of protocols across labs to support reproducibility and establish common understanding of the need for standardization (iii) Acknowledging and respecting that *originality* and *novelty* are at the core of research activities, the research community should explore mapping the overall landscape of standardization, identify areas for collaboration through projects and domestic/international conferences, and strive to consider making agreed-upon points, adoption criteria for conference presentations, and journal publications to promote harmonization

Regarding the first point, advancing R&D informed by technological trends as well as policy and societal developments, the study stressed the importance of the research community’s engagement in forward-looking activities, such as horizon-scanning, to understand technological trends, societal needs and values both domestically and internationally, and environmental factors surrounding the technology, such as rules. Research activities should be conducted with such insights, consciously identifying challenges through backcasting while considering social implementation. Such considerations should be pursued at the project, academic, and international levels. Regarding the second point, engaging in proactive deliberation to ensure safety and security, this paper proposes the following: (i) implementation of safety/security *by design* from the development stage, ensuring that data are properly acquired and stored from the outset, and development of regulatory science. Furthermore, these activities should be integrated into educational curricula and prioritized in discussions within academic societies; (ii) Engage in technological development to enhance safety and security, such as developing biological containment technologies that reduce risks through technical means; and (iii) When encountering their potential concerns or negative impacts, researchers themselves should raise concerns. The entire R&D community should engage in discussions with relevant stakeholders to address these issues. It is also important to engage with government agencies and international organizations, as necessary. Regarding the third point, contributing to discussions on standardization, the paper raised the following topics for consideration: (i) Integrate data standards (e.g., data sharing meeting FAIR principles), quality management, and LCA fundamentals into graduate curricula to provide training in standards from the early stages of research; (ii) Establish a shared understanding that a certain level of standardization is necessary for reproducibility and sharing of results among laboratories and develop and promote *bottom-up initiatives* for sharing protocols between laboratories; and (iii) Acknowledging and respecting that *originality* and *novelty* are at the core of research activities, the research community should explore mapping the overall landscape of standardization, identify areas for collaboration through projects and domestic/international conferences, and strive to consider making agreed-upon points, adoption criteria for conference presentations, and journals to promote harmonization.

These considerations require the mobilization of the expertise of researchers across different fields. In Japan, as noted in Section 2.1, several initiatives have begun to emerge, but these are sporadic and primarily driven within certain research projects and academic societies. Some programs, such as *the Frontier Development Program for Genome Editing* at Hiroshima University, have incorporated some ELSI lectures into their curricula. However, such efforts have not yet been integrated into nationwide education or curriculum frameworks. To foster talent and workforce in this area, investment and the development of supportive environments are essential. The necessity for such a multidisciplinary curriculum is being discussed, for example, through an international consortium established by UNESCO in collaboration with the Thai government. Systematically developing and structuring these elements into curricula is crucial. Furthermore, embedding these activities within the broader R&D process and developing them as personal initiatives is vital. Currently, there is no established mechanism within academia for evaluating contributions to regulatory science, standardization, and societal implementation. Therefore, the mechanisms that promote such activities must also be considered, such as making them eligible for awards in papers or conferences (the iGEM^41^ initiative is a good example of this) or setting them as specific conditions for journal acceptance.

Developing such mechanisms requires collaboration not only within academia but also with research funders, such as funding agencies and higher-level policymakers. In particular, regarding the codification and standardization of safety and security rules, top-down initiatives are essential to ensure their effectiveness. Therefore, policymakers must foster bottom-up initiatives and create and maintain an environment that enables R&D entities to consider ELSI/RRI. Furthermore, when concerns emerge through bottom-up channels, it is crucial to acknowledge them, clarify the current scientific uncertainties and the latest research status, and consider policy response pathways to implement the necessary measures (including the need for further research). For example, regarding “mirror life”, the United Kingdom Government Office for Science convened discussions at a roundtable meeting centered on experts such as the Government Chief Scientific Advisor, swiftly summarizing and presenting the risks and opportunities associated with mirror life^42^ (key researchers presented scientific evidence during these discussions). Currently, NEDO conducts requests for information^43^ to promote R&D. Establishing a mechanism to broadly ask what constitutes an ELSI issue may also be useful.

In summary, this study provides policy recommendations based on an analysis of policies and activities. While the analysis is grounded in Japanese policy, some aspects are applicable to other countries at the international level. Moving forward, it is necessary to elaborate on these recommendations through discussions with universities, academic societies, the government, funding agencies, industries, and other stakeholders.

## Footnotes

1 While calls for anticipatory governance are not new, organizations like the OECD have recently reaffirmed its importance in response to accelerating technological change (OECD, 2024).

2 Note that the term or concept of RRI is not explicitly used in Japanese science and technology policy (Okamura, 2021).

3 In Europe, Responsible Research and Innovation (RRI), which emerged under the seventh Framework Programme, Horizon 2020, no longer explicitly appears in Horizon Europe; however, it is said that the key elements of RRI have been embedded within the program. At the same time, in practice, monitoring under Horizon 2020 revealed significant diversity in the European RRI landscape and highlighted the need for mutual learning (Maier and Peter 2018).

4 The Operational and Evaluation Guidelines for the Moonshot R&D System (Cabinet Office (Japan), 2020b).

5 For example, for Goal 9, Increasing Peace of Mind and Vitality: Realizing a Mentally Rich and Dynamic Society, the call for proposals requires to include ELSI considerations and establish an ELSI response officer in their implementation plan, as specified in Appendix 2 Supplementary Notes by PD (Moonshot Goal 9).

6 Japan Science and Technology Agency (JST) website (in Japanese) Project Manager Recruitment (Moonshot-Type Research and Development Project). https://www.jst.go.jp/moonshot/koubo/202111/index.html [Accessed 31 August 2025].

7 Japan Science and Technology Agency (JST) website (in Japanese) Call for Proposals for Project Managers in Moonshot-Type Research and Development Program. https://www.jst.go.jp/moonshot/en/application/202111/index.html [Accessed 31 August 2025].

8 Furthermore, Goal 9 ELSI Guidelines have been established as a policy for research and development and social implementation that considers the ELSI aspects of Goal 9.Japan Science and Technology Agency (JST) website (in Japanese) https://www.jst.go.jp/moonshot/program/goal9/elsi/guideline.html Accessed 19 February 2026]).

9 Advanced Telecommunications Research Institute International (ATR) website. Internet of Brains (IoB). https://brains.link/en [Accessed 31 August 2025].

10 However, the extent to which ELSI is addressed varies depending on the strategic focus and timing of policy initiatives. In the most recent Bioeconomy Strategy published in 2024, the specific reference to ELSI has been relatively limited, confined primarily to the medical domain. However, even when ELSI is not explicitly mentioned, we can say that its substantive elements may still be covered—for instance, through an emphasis on rulemaking, international standardization, privacy protection, and data ethics.

11 The past example of examining the ELSI aspect of biotechnology includes consensus meetings on GM held in 2000 (Kobayashi, 2004).

12 Japan Science and Technology Agency (JST) website (in Japanese) ELSI Initiative in Genome-Related Technologies. https://www.jst.go.jp/ristex/internal_research/elsi/genome/[Accessed 31 August 2025].

13 Japan Science and Technology Agency (JST) website (in Japanese). ELSI Issue Map. https://www.jst.go.jp/ristex/internal_research/elsi/genome/genome_map.html [Accessed 31 August 2025].

14 Case studies include discussions that identified key issues through expert deliberation (Japan Science and Technology Agency (JST), 2024), as well as discussions utilizing citizen workshops and SF prototyping (JST-RISTEX Genome Ethics Research Group Case Study 2024 Report (JST-RISTEX, 2025)). Additionally, a future novel created using the SF prototyping method has a video available (Future Novel Created Using SF Prototyping Method JST-RISTEX youtube channel “Kind Dreams of Un-Kind” https://www.youtube.com/watch?v=BGjN4731ZUo [Accessed 31 August 2025]).

15 For example, embedding ELSI in research funding has not yet progressed to the point of allocating a portion of the budget.

16 For example, the Cabinet Office (Cabinet Office, 2020a) discusses concerns about bioethical issues arising from the birth of genome-edited babies, requiring policy responses from ELSI perspectives regarding genome editing.

17 For example, the Cabinet Office (Cabinet Office, 2019) calls for ELSI research on promoting social acceptance and public understanding of genome editing, reflecting its understanding of ELSI associated with social acceptance and promoting public understanding.

18 For example, the RRI tool developed under Europe’s FP7 provides a tool for RRI reflection items. It presents RRI approaches and methods tailored for each stakeholder group: policymakers, research and development, education, industry, and civil society.

19 RRI Tools Consortium website. RRI Self-Reflection Tool Blank Sheet. https://rri-tools.eu/documents/10184/265698/SRT-BlankSheet/56970b0c-49a4-401a-b69b-7ff4ea7fcf74 [Accessed 31 August 2025].

20 RRI Toolkit for Enterprises (Responsible Innovation Compass Consortium, 2019).

21 It is often stated that Japan wins in developing technology but loses in rulemaking. In response to this, policy discussions have increasingly emphasized the need for Japan to shift from being a *rule-taker* to becoming a *rule-maker*. For example, the METI website features the Certification System for Specific New Demand Development Business Activity Plans (OCEAN Project).

22 METI website (in Japanese). https://www.meti.go.jp/policy/economy/hyojun-kijun/katsuyo/ocean_project/index.html [Accessed 31 August 2025].

23 MHLW website (in Japanese). BSL-4 Facility Standards Based on the Infectious Diseases Control Law https://www.mhlw.go.jp/stf/seisakunitsuite/bunya/kenkou_iryou/kenkou/kekkaku-kansenshou/kijun_bsl4.html[Accessed 31 August 2025].

24 NITE website (in Japanese). https://www.nite.go.jp/nbrc/cultures/nbrc/use/specialset.html (September 2025 accessed)

25 NCBI Web page https://www.ncbi.nlm.nih.gov/(September 2025 accessed).

26 EMBL HP https://www.embl.org/(September 2025 accessed).

27 Representative standards for biomass feedstocks include ASTM E1755 (Ash), E1756 (Total Solids and Moisture), E872 (Volatile Matter), and ASTM E48 Subcommittee and ISO 16948:2015 (Elemental Composition of Solid Biofuels), ISO 17225 (Specifications and Classification of Solid Biofuels) as international standards.

28 ASTM E1755-01: Standard Test Method for Ash in Biomass. ASTM International website. https://store.astm.org/e1755-01.html [Accessed 31 August 2025].

29 ASTM E1756-08r20: Standard Test Method for Determination of Total Solids in Biomass.ASTM International website. https://store.astm.org/e1756-08r20.html [Accessed 31 August 2025].

30 ASTM E0872-82r19: Standard Test Method for Moisture Analysis of Particulate Biomass. ASTM International website. https://store.astm.org/e0872-82r19.html [Accessed 31 August 2025].

31 Committee E48 on Bioenergy and Industrial Chemicals from Biomass website. https://www.astm.org/membership-participation/technical-committees/committee-e48 [Accessed 31 August 2025].

32 Solid biofuels—Determination of total content of carbon, hydrogen and nitrogen. International Organization for Standardization website. https://www.iso.org/standard/58004.html [Accessed 31 August 2025].

33 ISO 17225-1:2021: Solid biofuels—Determination of total content of carbon, hydrogen and nitrogen. International Organization for Standardization website. https://www.iso.org/standard/58004.html [Accessed 31 August 2025].

34 ISO 17225-2:2021: Solid biofuels—Fuel specifications and classes—Part 2: Graded wood pellets. International Organization for Standardization website. https://www.iso.org/standard/76088.html [Accessed 31 August 2025].

35 NEDO website (in Japanese). About the Green Innovation Fund. https://green-innovation.nedo.go.jp/about/[Accessed 31 August 2025].

36 EC website, European Platform on LCA | EPLCA. https://eplca.jrc.ec.europa.eu/lifecycleassessment.html [Accessed 31 August 2025].

37 There are still other challenges. Conventional LCA is fundamentally based on material flows. In synthetic biology, genetically modified organisms are the primary raw materials. This makes it difficult to predict the environmental impacts of their use, such as the evolution of the organisms themselves and effects on the external environment (e.g., horizontal gene transfer). Consequently, there is a fundamental criticism that LCA, which simply calculates environmental impacts from material transformation, does not function effectively (Seager et al., 2017).

38 Cell Press website. Cell Press: STAR Protocols. https://www-cell-com.utokyo.idm.oclc.org/star-protocols/home [Accessed 31 August 2025].

39 MyJoVE Corporation website. Peer Reviewed Scientific Video Journal Article Protocols | JoVE. https://www.jove.com/[Accessed 31 August 2025].

40 protocols.io website. Bring structure to your research. https://www.protocols.io [Accessed 31 August 2025].

41 iGEM is a student competition in engineering biology, and its evaluation criteria go beyond scientific aspects to encompass a broad range of dimensions, including the integration of human practices, safety and security (Millet et al., 2019), education, inclusivity, entrepreneurship, and sustainable development impact. It is a highly valuable initiative in that it encourages students (including high school students) to engage in such activities from an early age (iGEM Foundation, 2024).

42 Government Office for Science website. Mirror life. https://www.gov.uk/government/publications/mirror-life/mirror-life [Accessed 31 August 2025].

43 NEDO also issues requests for information for project manager support. [New Energy and Industrial Technology Development Organization (NEDO, 2021)], New Energy and Industrial Technology Development Organization (NEDO) website (in Japanese). Request for Information (RFI) Regarding Support for Project Managers through the Application of Mathematical, Humanities, and Social Sciences in the Moonshot-Type Research and Development Program. https://www.nedo.go.jp/news/other/Z1MS_00004.html [Accessed 31 August 2025].
